# Evaluating a calcium-aware kernel for CT CAC scoring with varying surrounding materials and heart rates: a dynamic phantom study

**DOI:** 10.1007/s00330-021-08076-5

**Published:** 2021-05-28

**Authors:** Niels R. van der Werf, Ronald Booij, Bernhard Schmidt, Thomas G. Flohr, Tim Leiner, Joël J. de Groen, Daniël Bos, Ricardo P. J. Budde, Martin J. Willemink, Marcel J. W. Greuter

**Affiliations:** 1 Department of Radiology, University Medical Center Utrecht, Utrecht, The Netherlands; 2 Department of Radiology & Nuclear Medicine, Erasmus University Medical Center, Rotterdam, The Netherlands; 3 Computed Tomography, Siemens Healthineers, Forchheim, Germany; 4 Department of Epidemiology, Erasmus University Medical Center, Rotterdam, The Netherlands; 5 Department of Radiology, University of Groningen, University Medical Center Groningen, Groningen, The Netherlands; 6 Department of Radiology, Stanford University School of Medicine, Stanford, CA USA; 7 Department of Robotics and Mechatronics, University of Twente, Enschede, The Netherlands

**Keywords:** Tomography, x-ray computed, Coronary artery disease, Diagnostic imaging, Radiation dosage

## Abstract

**Objectives:**

The purpose of this study was twofold. First, the influence of a novel calcium-aware (Ca-aware) computed tomography (CT) reconstruction technique on coronary artery calcium (CAC) scores surrounded by a variety of tissues was assessed. Second, the performance of the Ca-aware reconstruction technique on moving CAC was evaluated with a dynamic phantom.

**Methods:**

An artificial coronary artery, containing two CAC of equal size and different densities (196 ± 3, 380 ± 2 mg hydroxyapatite cm^−3^), was moved in the center compartment of an anthropomorphic thorax phantom at different heart rates. The center compartment was filled with mixtures, which resembled fat, water, and soft tissue equivalent CT numbers. Raw data was acquired with a routine clinical CAC protocol, at 120 peak kilovolt (kVp). Subsequently, reduced tube voltage (100 kVp) and tin-filtration (150Sn kVp) acquisitions were performed. Raw data was reconstructed with a standard and a novel Ca-aware reconstruction technique. Agatston scores of all reconstructions were compared with the reference (120 kVp) and standard reconstruction technique, with relevant deviations defined as > 10%.

**Results:**

For all heart rates, Agatston scores for CAC submerged in fat were comparable to the reference, for the reduced-kVp acquisition with Ca-aware reconstruction kernel. For water and soft tissue, medium-density Agatston scores were again comparable to the reference for all heart rates. Low-density Agatston scores showed relevant deviations, up to 15% and 23% for water and soft tissue, respectively.

**Conclusion:**

CT CAC scoring with varying surrounding materials and heart rates is feasible at patient-specific tube voltages with the novel Ca-aware reconstruction technique.

**Key Points:**

*• A dedicated calcium-aware reconstruction kernel results in similar Agatston scores for CAC surrounded by fatty materials regardless of CAC density and heart rate.*

*• Application of a dedicated calcium-aware reconstruction kernel allows for radiation dose reduction.*

*• Mass scores determined with CT underestimated physical mass.*

## Introduction

Coronary artery calcifications (CACs), as detected by non-contrast cardiac computed tomography (CT), are a strong predictor for future adverse cardiovascular events [1–3]. With CT, CAC is traditionally quantified according to the Agatston scoring standard [4]. Other CAC scores, such as the mass score, were developed to decrease shortcomings of the Agatston methodology [5–10]. The number of CT CAC assessments in clinical practice has increased substantially, which in turn increased the cumulative radiation exposure to patients undergoing these exams [11]. In order to standardize CT CAC scoring across different types of CT equipment, a setting of 120 kVp in combination with 3-mm slice thickness is recommended [12]. On the other hand, the most efficient way to reduce radiation dose for CT CAC imaging is to decrease the peak tube voltage (kVp) to values below 120 kVp. However, adjusting peak tube voltage will change Agatston scores and has therefore shown to be difficult to implement [4, 10, 13].

To address this tradeoff, a novel calcium-aware (Ca-aware) reconstruction technique was recently introduced by one of the main CT manufacturers [14]. The aim of this Ca-aware reconstruction kernel is to minimize the previously shown kVp-induced variability of Agatston scores in order to allow for acquisitions at patient-specific lower tube voltages without affecting CAC scores [15, 16]. This novel reconstruction kernel is optimized for CAC surrounded by fat, as found in vivo where the arteries are embedded in the epicardial fat. In the reconstruction process, bone and calcium are identified after which a voltage-dependent lookup table is used to convert CT numbers to values which correspond to a tube potential of 120 kVp. Ideally, this will yield CAC scores obtained at reduced tube voltages that are equivalent to traditional scores that would have been obtained with 120-kVp acquisitions. Changing the resulting tube voltage-dependent Hounsfield unit (HU) values of CAC to their 120-kVp values may enable the use of Agatston calcium scoring methodology independent of tube voltage. For acquisitions with tube voltages below 120 kVp, or with added filtration, this may enable decreased radiation dose while maintaining unchanged CAC scores.

An important confounder of CAC scores is residual motion of coronary arteries during image acquisition [17–20]. Motion artifacts can increase or decrease CAC scores, depending on the density of the calcification [20]. While recent studies have assessed the influence of the novel Ca-aware reconstruction technique on both stationary calcifications and in patients, the effect of this reconstruction technique on CAC scores of moving calcifications of different densities at varying heart rates remains unknown [21–23].

Against this background, we formulated the following two aims. Our first aim was to assess the influence of the Ca-aware reconstruction technique on CAC scores of calcifications with different densities surrounded by a variety of patient equivalent tissues. Second, the influence of the Ca-aware reconstruction technique on moving calcifications was assessed with a dynamic anthropomorphic phantom.

## Materials and methods

### Phantom

**Fig. 1:**
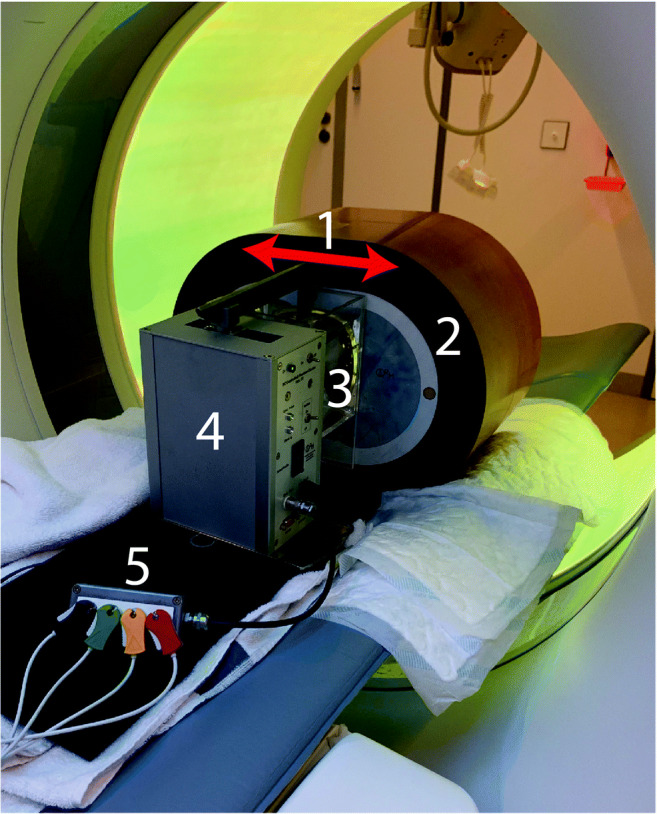
Overview of phantom setup, with the robotic arm moving a coronary artery along the direction indicated by the red arrow (1), in the anthropomorphic thorax phantom (2), within the fillable compartment (3). Movement is generated with the Sim2D robot (4), which also provides an electrocardiogram output (5) to ensure data acquisition during linear motion of the artificial coronary artery

An anthropomorphic thorax phantom (QRM-thorax, QRM) containing artificial lungs, a spine, and a shell of soft tissue equivalent material was used (Fig. 1). An extension ring of fat equivalent material (QRM-Extension Ring, QRM) was used to increase the phantom dimensions to 400 × 300 mm, similar to the dimensions of an averaged-sized patient [15].

A fillable compartment was placed within the cylindrical hole in the center of the thorax phantom, in which an artificial coronary artery was linearly translated at constant velocities of 0, 10, 20, and 30 mm/s with the use of a robotic arm (Sim2D, QRM). The artificial coronary artery contained both a low- and medium-density calcification of 196 ± 3 and 380 ± 2 mg hydroxyapatite (HA) cm^−3^, respectively. Both calcifications were equal in size: 5.0 ± 0.1 mm in diameter, with a length of 10.0 ± 0.1 mm. The movement was in a horizontal plane, perpendicular to the scan direction. The velocities of the artificial coronary artery corresponded to the average movement of in vivo coronary arteries during the scan phase at 0, < 60, 60–75, and > 75 beats per minute (bpm) [18]. To ensure that only constant velocities were present during the scan phase, the robotic arm was synchronized with the CT system during acquisition with the use of the electrocardiography trigger output.

### Data acquisition and reconstruction

Raw data was acquired with a vendor recommended protocol for CT CAC scoring at 120 kVp on a state-of-the-art CT system (SOMATOM Force, Siemens Healthineers) (Table 1). Images were reconstructed with filtered back projection (FBP), using the standard CAC scoring technique (kernel Qr36f), and the Ca-aware reconstruction technique (kernel Sa36). Furthermore, besides the standard 120-kVp acquisition, two other acquisitions were performed. First, data was acquired based on automatic tube voltage selection (CARE kV, Siemens Healthineers) for the water equivalent thickness of the phantom. Second, a dedicated CAC tin-filtration protocol was used. For all protocols, tube current was adjusted according to automatic tube current modulation (CARE Dose4D, Siemens Healthineers) (Table 1). The quality reference was set at 80 mAs/rotation, with the dose optimization slider on position 5 (calcium/bone). Due to a limitation in tube current with automatic tube voltage selection for the used phantom size and 100SnkVp, a tube potential of 150Sn kVp was manually selected. To increase sample size and precision, each acquisition was repeated five times for each heart rate. Between each scan, the phantom was manually translated and rotated by approximately 2 mm and 2 degrees, respectively.

**Table 1:** Acquisition and reconstruction parameters for the reference, reduced kVp, and tin-filtration scans

Parameter	Reference	Reduced kVp	Tin filtration
Acquisition mode	Sequential	Sequential	Sequential
Ref. tube voltage (kVp)	120	120	100Sn
Ref. tube current product^1^ (mAs/rot)	80	80	534
Tube voltage (kVp)	120	100^2^	150Sn^2^
Tube current (mAs/rotation)	100^3^	148^3^	136^3^
Collimation (mm)	2 × 96 × 0.6	2 × 96 × 0.6	2 × 96 × 0.6
Rotation time (s)	0.25	0.25	0.25
Temporal resolution (ms)	66	66	66
Slice thickness (mm)	3	3	3
Slice increment (mm)	1.5^4^	1.5^4^	1.5^4^
Kernel	Qr36f/Sa36f^5^	Qr36f/Sa36f^5^	Qr36f/Sa36f^5^
Reconstruction	FBP	FBP	FBP
Matrix	512 × 512	512 × 512	512 × 512
Field of view (mm)	220	220	220
CTDIvol (mGy)	3.93	3.45	2.47

^1^Default quality reference tube current, with dose optimization slider at position 5 (calcium/bone)

^2^Automatically selected based on phantom size

^3^Based on water equivalent thickness of used phantom setup

^4^Standard for calcium scoring with Siemens Healthineers equipment

^5^Ca-aware reconstruction kernel (Sa36f) used to compare results with reference reconstruction kernel (Qr36f)

*Ref* reference, *rot* rotation, *CTDIvol* CT dose index volume, *FBP* filtered back projection

In addition to Agatston scores, we obtained mass scores by acquiring additional images according to each of the three abovementioned protocols with a static cardiac calcification insert that included calcium calibration rods (CCI, QRM). These reconstructions were used to calculate the mass calibration factor for each of our protocols, according to the methodology described by McCollough et al [13].

**Fig. 2:**
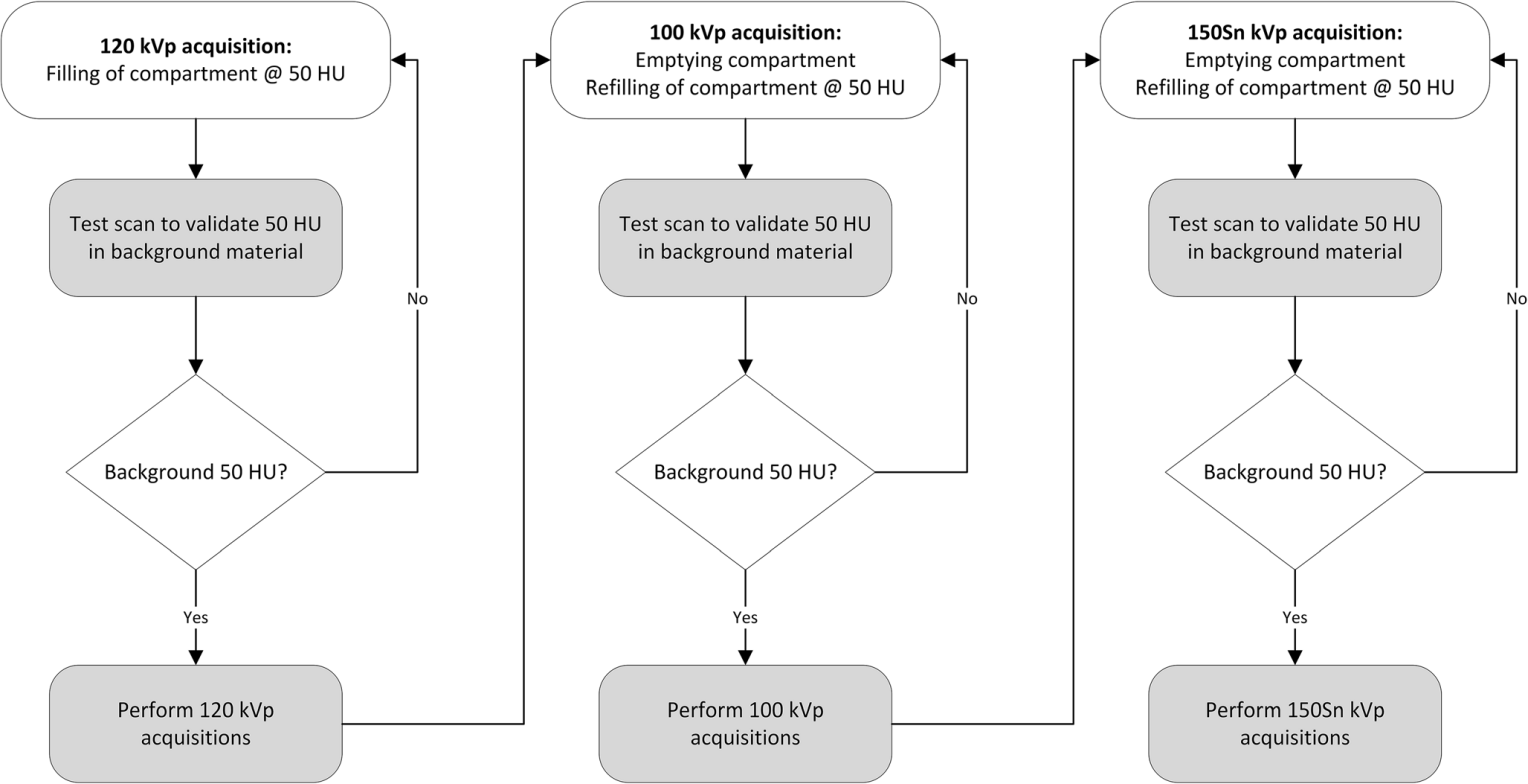
Flowchart which describes the methodology to account for the tube voltage dependency of the used iodine-water mixture to resemble soft tissue

The fillable compartment, placed in the anthropomorphic phantom, was used to subsequently acquire data with three materials, adjacent to the artificial coronary artery. These materials resembled fat (−100 HU, water-ethanol mixture), water (0 HU), and soft tissue (50 HU, water-iodine contrast agent (Iodixanol) mixture). To account for the tube voltage dependency of our mixtures, for each acquisition (120, 100, 150Sn kVp), the compartment was filled for that specific tube voltage, as indicated by the flowchart in Fig. 2 for the soft tissue acquisitions. Prior to every acquisition, each mixture was manually stirred to prevent curdling of the liquids. In addition, due to the usage of the robotic arm of the dynamic phantom, the mixture was stirred continuously, except during the 0-mm/s acquisitions.

### Data analysis

CAC scores were determined from the resulting reconstructed images using a previously validated, in-house developed Python script (Python version 3.7) [21]. A calcium scoring threshold of 130 HU was used for all reference and Ca-aware reconstruction kernel data. For the reduced-kVp acquisition in combination with the standard kernel, a threshold of 147 HU was used, as described previously [16]. As such an adapted threshold was not available for 150Sn kVp acquisitions, the regular 130 HU threshold was used. For all acquisitions, mean Agatston and mass scores and standard deviation (SD) were calculated from the five repeated measurements for each combination of heart rate, background material, and acquisition protocol. In addition, an Agatston score was calculated for each reconstruction in a uniform background region-of-interest, without any calcium content. This resulted in a background Agatston score (BAS), which is only larger than zero for high image noise levels, as previously described by Booij et al [21].

For each heart rate, Agatston scores of the reduced-kVp and tin-filtration acquisition were compared to the 120-kVp reference. Differences in Agatston score ≥ 10% were deemed to be relevant. Resulting mass scores were compared to the physical mass of the calcifications. Again, relevant differences were set at ≥ 10%.

## Results

### Background material and radiation dose

Automatic tube voltage selection, based on the water equivalent thickness of the phantom, resulted in a tube voltage of 100kVp, while 150Sn kVp was manually selected. For these protocols, this resulted in a radiation dose of 3.45 and 2.83 mGy, respectively. In comparison with the radiation dose of 3.93 mGy for the reference acquisition, this was a reduction of 12% and 28% for the reduced-kVp and tin-filtration acquisitions, respectively.

On average, overall velocities and repetitions, background material mean HU (± SD) and image noise values for fat, water, and soft tissue equivalent material, are shown in Table 2. Noise levels from the tin-filtration protocol resulted in BAS > 0 for all acquisitions.

**Table 2:** Background material mean (mean ± SD) and noise (mean ± SD) for all combinations of tube potential, reconstruction kernel, and background material, on average for all used velocities and repetitions

Tube potential (kVp)	Kernel	Fat		Water		Soft tissue	
		Mean	Noise	Mean	Noise	Mean	Noise
120	Standard	−94.4 ± 0.2	19.9 ± 0.7	0.0 ± 0.4	23.8 ± 0.3	51.3 ± 0.4	25.0 ± 0.4
	Ca-aware	−93.8 ± 0.3	19.9 ± 0.7	0.8 ± 0.4	23.8 ± 0.4	52.3 ± 0.4	25.0 ± 0.4
100	Standard	−99.0 ± 0.3	21.0 ± 1.1	−1.7 ± 0.3	25.3 ± 0.4	51.8 ± 0.3	26.3 ± 0.6
	Ca-aware	−98.5 ± 0.3	21.2 ± 1.0	−1.0 ± 0.3	25.4 ± 0.4	52.3 ± 0.3	26.2 ± 0.5
150Sn	Standard	−84.5 ± 0.4	23.9 ± 0.6	2.4 ± 0.4	27.6 ± 0.6	49.7 ± 0.5	28.5 ± 0.5
	Ca-aware	−84.2 ± 0.3	23.7 ± 0.3	2.9 ± 0.4	27.8 ± 0.6	54.6 ± 0.6	31.4 ± 0.7

### Influence of background material on Agatston scores

**Fig. 3:**
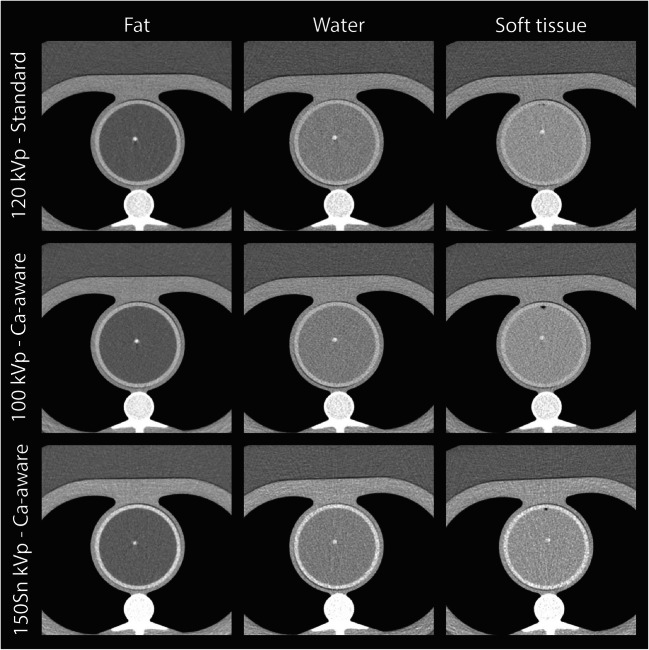
Overview of representative images of the low-density calcifications for different combinations of acquisition and reconstruction parameters, and background material, for > 75 bpm

**Fig. 4:**
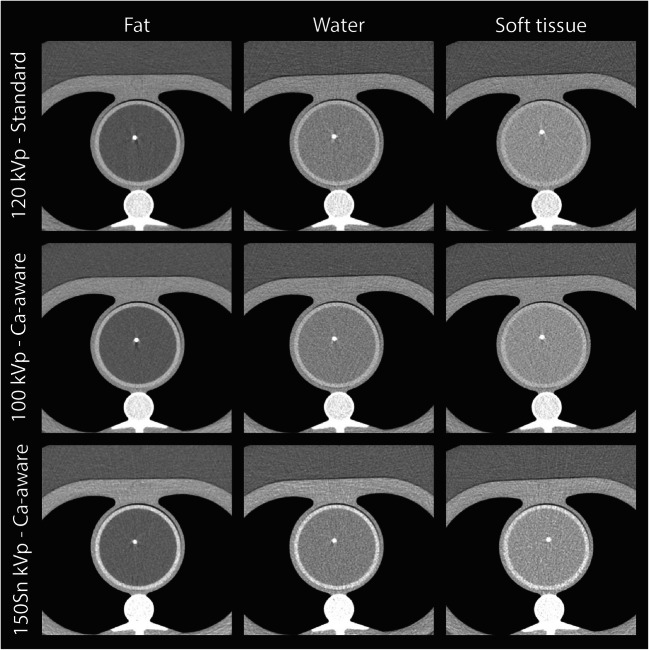
Overview of representative images of the medium-density calcifications for different combinations of acquisition and reconstruction parameters, and background material, for > 75 bpm

**Fig. 5:**
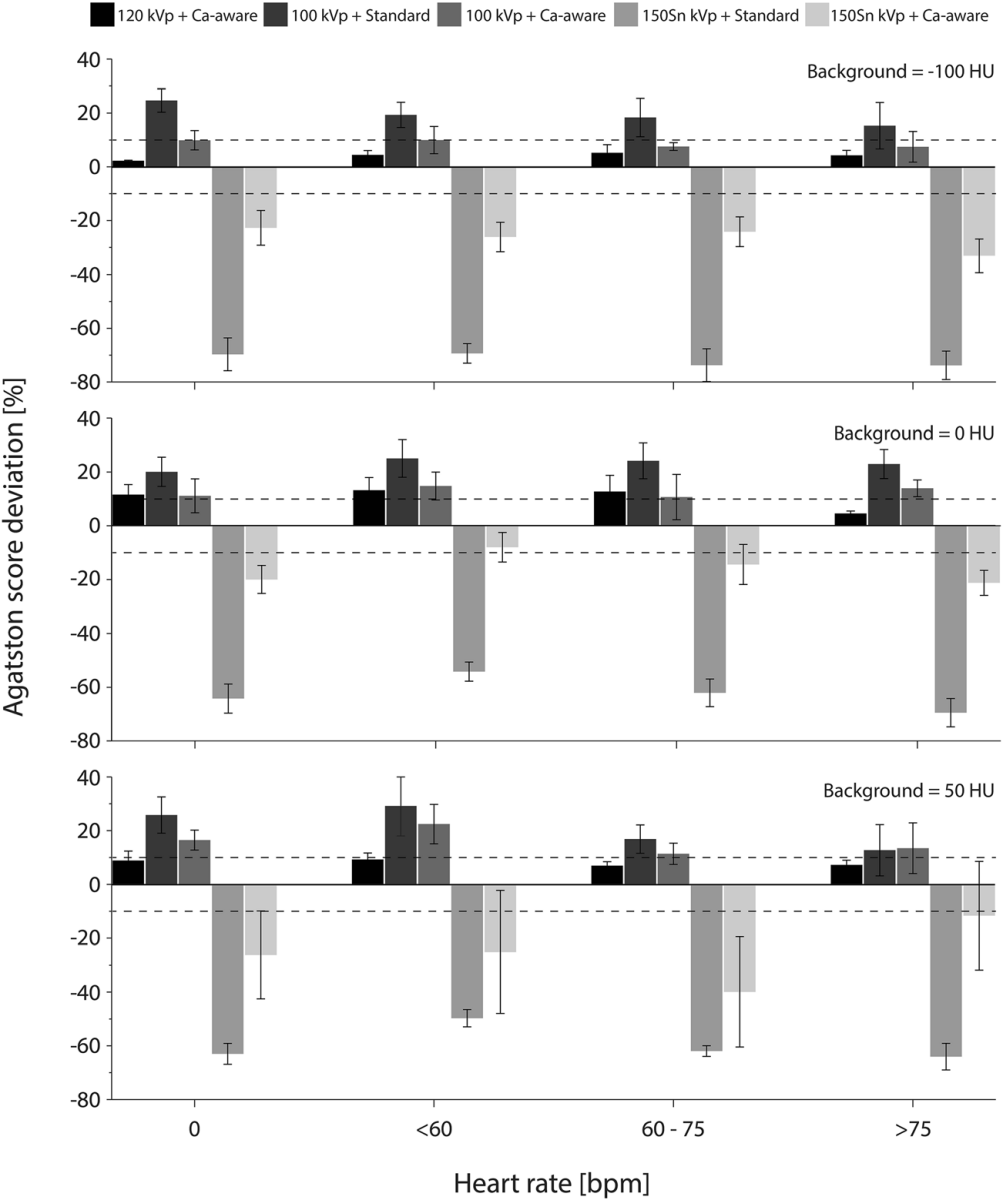
Deviations in low-density CAC Agatston score from the reference (for each heart rate: 120 kVp + standard reconstruction kernel) for different heart rates and combinations of tube voltage (kVp) and reconstruction. Results are shown for three background materials: fat (−100 HU, top), water (0 HU, middle), and soft tissue (50 HU, bottom). Clinically relevant differences, at ≥ ± 10%, are indicated with dashed lines

**Fig. 6:**
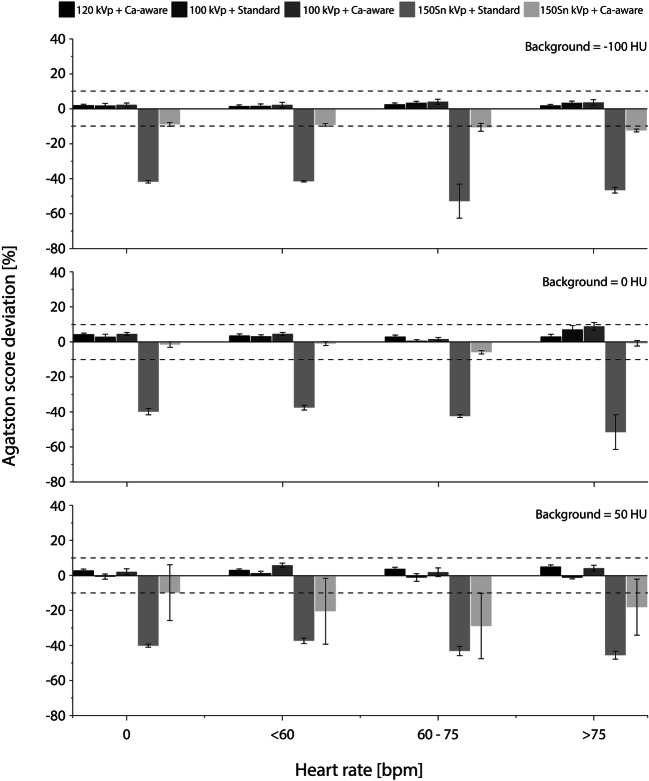
Deviations in medium-density CAC Agatston score from the reference (for each heart rate: 120 kVp + standard reconstruction kernel) for different heart rates and combinations of tube voltage (kVp) and reconstruction kernel. Results are shown for three background materials: fat (−100 HU, top), water (0 HU, middle), and soft tissue (50 HU, bottom). Clinically relevant differences, at ≥ ± 10%, are indicated with dashed lines

Representative images for the highest heart rate are shown in Figs. 3 and 4, for the low- and medium-density calcification, respectively. These figures show a reduced detectability for decreased CAC density and increased surrounding material HU. Agatston scores resulting from the different acquisition and reconstruction protocols are shown in Figs. 5 and 6, again for the low- and medium-density calcification, respectively. From these data, it is clear that the standard reconstruction technique leads to clinically relevant differences for all background materials and heart rates for the low-density CAC. With the Ca-aware reconstruction technique, Agatston scores are comparable with the reference. For each velocity, deviations from the reference (FBP with standard kernel) were smaller for the medium-density calcification compared to the low-density calcification. Further, almost all tin-filtration acquisitions led to > 10% differences in comparison with the reference acquisition for all three background materials.

For all heart rates, Agatston scores for the calcifications submerged in fat-like background material were comparable to the reference, for the reduced-kVp acquisition with Ca-aware reconstruction kernel. For water and soft tissue, medium-density Agatston scores were again comparable to the reference for all heart rates. However, low-density Agatston scores showed clinically relevant deviations for all heart rates, when the calcification was submerged in water or soft tissue–like material.

### Comparison with physical mass

Physical mass underestimated low-density calcifications by approximately 50% for all reference acquisition and reconstruction settings (Tables 3 and 4). These underestimations of the physical mass changed to −50%, −41%, and −34% for the reduced-kVp on average for all heart rates, for fat, water, and soft tissue adjacent material, respectively. For the tin-filtration protocol, the underestimation of HU values ranged −65%, −53%, and −31%. Medium-density calcification mass scores underestimated physical mass by −29%, −18%, and −30%, again on average for all heart rates, for fat, water, and soft tissue circumjacent material, respectively. These values changed to −31%, −20%, and −9% for the kVp-reduced protocol, and −36%, −19%, and −10% for the tin-filtration protocol.

**Table 3:** Percent difference with physical mass of the low-density calcification in mean ± SD for all acquisition, reconstruction, background material, and heart rate parameters. To calculate the percentage difference, the numerator was the mass score, and the denominator was the physical mass (38 mg)

Background material	Heart rate	120 kVp		100 kVp		150Sn kVp	
		Standard	Ca-aware	Standard	Ca-aware	Standard	Ca-aware
Fat	0	−47.9 ± 2.2	−47.8 ± 2.3	−45.1 ± 2.0	−46.2 ± 1.8	−73.7 ± 5.4	−63.0 ± 4.1
	< 60	−48.9 ± 1.7	−48.8 ± 1.5	−46.0 ± 3.0	−47.2 ± 2.9	−72.8 ± 2.5	−63.0 ± 2.7
	60–75	−53.5 ± 0.6	−53.5 ± 0.6	−51.1 ± 1.6	−52.6 ± 1.6	−77.2 ± 8.8	−64.9 ± 3.5
	> 75	−55.8 ± 1.8	−55.8 ± 1.8	−54.7 ± 3.5	−56.0 ± 3.5	−78.7 ± 5.3	−69.8 ± 3.8
Water	0	−39.5 ± 2.9	−38.7 ± 2.9	−38.0 ± 2.3	−39.0 ± 2.3	−62.3 ± 3.7	−49.9 ± 2.6
	< 60	−42.7 ± 2.3	−42.2 ± 2.4	−37.7 ± 1.1	−38.8 ± 1.0	−59.5 ± 4.1	−47.5 ± 3.7
	60–75	−41.4 ± 2.5	−40.7 ± 2.1	−39.1 ± 1.6	−40.7 ± 1.5	−65.3 ± 7.4	−53.4 ± 6.7
	> 75	−47.4 ± 2.0	−46.8 ± 1.8	−42.9 ± 0.7	−44.2 ± 1.3	−74.3 ± 8.5	−57.7 ± 3.0
Soft tissue	0	−62.3 ± 3.7	−49.9 ± 2.6	−33.5 ± 2.3	−32.4 ± 2.0	−28.5 ± 2.6	−29.8 ± 2.9
	< 60	−59.5 ± 4.1	−47.5 ± 3.7	−39.3 ± 6.4	−37.5 ± 5.9	−26.3 ± 4.0	−27.6 ± 4.1
	60–75	−74.3 ± 8.5	−57.7 ± 3.0	−35.3 ± 4.5	−33.4 ± 4.2	−30.5 ± 3.6	−33.6 ± 2.6
	> 75	−74.3 ± 8.5	−57.7 ± 3.0	−35.3 ± 4.5	−33.4 ± 4.2	−30.5 ± 3.6	−33.6 ± 2.6

**Table 4:** Percent difference with physical mass of the medium-density calcification in mean ± SD for all acquisition, reconstruction, background material, and heart rate parameters. To calculate the percentage difference, the numerator was the mass score, and the denominator was the physical mass (74 mg)

Background material	Heart rate	120 kVp		100 kVp		150Sn kVp	
		Standard	Ca-aware	Standard	Ca-aware	Standard	Ca-aware
Fat	0	−26.7 ± 1.2	−27.3 ± 1.1	−28.2 ± 0.9	−29.1 ± 1.0	−36.1 ± 1.9	−34.0 ± 1.5
	< 60	−27.4 ± 0.4	−28.1 ± 0.3	−29.4 ± 1.1	−30.3 ± 1.0	−36.4 ± 1.1	−34.1 ± 1.2
	60–75	−29.3 ± 1.4	−29.9 ± 1.4	−30.7 ± 1.3	−31.7 ± 1.4	−49.9 ± 19.5	−37.5 ± 2.6
	> 75	−34.0 ± 0.8	−34.5 ± 0.8	−33.1 ± 1.6	−34.2 ± 1.5	−42.8 ± 2.7	−40.0 ± 2.2
Water	0	−17.4 ± 1.7	−17.4 ± 1.7	−18.3 ± 1.9	−19.1 ± 2.1	−21.1 ± 1.3	−16.3 ± 0.7
	< 60	−17.7 ± 1.6	−17.6 ± 1.7	−18.5 ± 1.3	−19.2 ± 1.4	−22.0 ± 1.0	−18.4 ± 1.2
	60–75	−18.7 ± 1.0	−18.7 ± 1.1	−21.2 ± 1.1	−22.1 ± 1.1	−26.2 ± 2.7	−21.6 ± 2.8
	> 75	−20.1 ± 2.1	−20.1 ± 2.0	−18.8 ± 1.9	−19.9 ± 1.9	−38.9 ± 23.5	−21.1 ± 2.4
Soft tissue	0	−21.1 ± 1.3	−16.3 ± 0.7	−10.9 ± 0.9	−10.6 ± 0.9	−11.8 ± 0.8	−12.6 ± 0.7
	< 60	−22.0 ± 1.0	−18.4 ± 1.2	−12.6 ± 1.1	−12.2 ± 1.2	−11.4 ± 0.5	−12.1 ± 0.5
	60–75	−38.9 ± 23.5	−21.1 ± 2.4	−7.0 ± 1.9	−5.6 ± 2.1	−5.5 ± 2.9	−7.3 ± 2.2
	> 75	−38.9 ± 23.5	−21.1 ± 2.4	−7.0 ± 1.9	−5.6 ± 2.1	−5.5 ± 2.9	−7.3 ± 2.2

## Discussion

The main finding of this study is that the Ca-aware reconstruction kernel performs well for a patient-specific tube voltage acquisition protocol (12% radiation dose reduction), for medium-density CAC, irrespective of CAC adjacent material or heart rate. However, in the presence of low-density CAC, substantial deviations in Agatston scores were observed, when calcifications were surrounded by water (up to 15%) or soft tissue (up to 22%) equivalent material, irrespective of heart rate. Furthermore, the tin-filtration protocol also led to substantial deviations in Agatston scores for low-density calcifications, for most combinations of heart rates and surrounding tissue. Furthermore, noise levels for this protocol were high, leading to BAS > 0. Finally, mass scores as assessed by CT underestimated the true physical mass.

To the best of our knowledge, this study is the first to systematically assess the performance of a novel Ca-aware reconstruction kernel for different CAC surrounding materials, CAC densities, and heart rates. In general, and especially for the low-density calcification, reduced-kVp acquisitions resulted in increased Agatston scores. This is expected, as the energy dependence of CT numbers of the surrounding material (fat/water/soft tissue) is different from the energy dependence of the CT number of calcium, as previously described by Jakobs et al [24]. Because of the phenomenon, the detectability of calcium is increased, especially at the margins of CAC where voxels might be just below the calcium scoring threshold for 120kVp. In turn, as more CAC is detected, more voxels are taken into account by the Ca-aware reconstruction kernel for its recalculation to 120-kVp HU values [14].

Our results are in line with a phantom study by Booij et al, who demonstrated that the consistency of CT numbers was reduced for low-density CAC when comparing CT numbers from reduced tube voltage acquisitions reconstructed with the Ca-aware kernel, and CT numbers from routine protocols [21]. In their study, however, a base-material correction factor was provided by the CT manufacturer [14]. This correction factor was necessary to account for the tube voltage dependency of all materials other than water. The usage of this correction factor hampers direct comparison with our results, as this artificial step was necessary due to the nature of the used phantom in their study, which might have influenced resulting Agatston scores.

In addition, two patient studies have been carried out by Vingiani et al [22, 23]. Both studies showed the feasibility of the Ca-aware reconstruction kernel, in combination with patient-specific tube voltages, where one study considered spectral beam shaping with tin filtration. Comparison of results is hampered by the fact that for their first study, 100 kVp with tin filtration was applied, whereas in our study a tube voltage of 150 kVp with tin filtration was manually selected for the phantom [22]. In the other study by Vingiani et al, forty-three patients were imaged with both 120 kVp and an individualized tube voltage [23]. A high concordance in Agatston scores between both scans was found. Since the density of these CAC and the HU of the CAC surrounding material are unknown, it is not known if these results are in line or contradictory to our results.

In line with previous studies, we found that CT generally underestimates the physical mass of the low-density calcifications by approximately 50% for all reference acquisition and reconstruction settings [20].

Our study has some limitations that merit consideration. First, this was an in vitro study, with artificial CAC containing coronary arteries and artificial background material. Nevertheless, the coronary arteries were translated in an anthropomorphic chest phantom at velocities which were observed in in vivo studies [18]. Also, the mass of the calcifications was in the range which is observed in patients [25]. Second, movement of the artificial coronary arteries was only linear and in the horizontal plane, while in vivo complex movements in three dimensions are observed. As the actual scan phase of a CAC scan is only 104 ms, based on the total detector coverage and rotation time, we approximate that the addition of 3D movement would only result in minor changes in our results. Third, the materials used to simulate in vivo CAC circumjacent tissue are artificial. However, the linear attenuation coefficient of the materials is only used for the Ca-aware reconstruction kernel. The exact chemical composition is therefore irrelevant for the current analysis, as we changed the ratio of our mixtures for each tube voltage to ensure stable background material HU. Fourth, tube current limitations of the dedicated CAC tin-filtration protocol led to the usage of semi-automatic tube voltage selection for our tin-filtration acquisitions and a manual selection of 150Sn kVp. However, calcium contrast is inherently low for this hardened X-ray spectrum, resulting in reduced CAC detectability and quantification results. Although available to be manually selected by CT radiographers, this 150Sn protocol is not recommended by the CT manufacturer for CAC scoring for large patients. In addition, a low-dose value was selected for the 150Sn kVp protocol which resulted in increased noise levels and may have affected the determination of the Agatston score of the calcifications and may have led to Agatston scores for a non-CAC containing ROI (BAS > 0). This means that the resulting Agatston scores for these acquisitions might be overestimated. However, for all combinations of surrounding material, CAC density, and heart rate, clinically relevant decrease in Agatston score was shown. This decrease should therefore be even larger, when noise levels were lower.

CT CAC scoring with varying surrounding materials and heart rates is feasible at patient-specific tube voltages with the novel Ca-aware reconstruction technique.

## Abbreviations

BAS: Background Agatston score
Bpm: Beats per minute
Ca-aware: Calcium aware
CAC: Coronary artery calcification
CT: Computed tomography
FBP: Filtered back projection
HA: Hydroxyapatite
HU: Hounsfield unit
kVp: Peak tube voltage
mGy: Milligray
SD: Standard deviation

## Declarations

### Guarantor

The scientific guarantor of this publication is Marcel Greuter.

### Statistics and biometry

No complex statistical methods were necessary for this paper.

### Methodology

• Prospective

• Experimental

• Performed at one institution
